# Atomization of Water Jet in Crossflow via High-Speed Photography

**DOI:** 10.1038/s41598-025-89276-7

**Published:** 2025-02-28

**Authors:** Benshuai Fu, Haiyan Xiao, Bingju Lu, Guanghua Li, Liping Qin

**Affiliations:** 1 School of Mechanical and Electrical Engineering, Harbin Engineering University, Harbin, 150001 China; 2 Henan Key Laboratory of Underwater Intelligent Equipment, Zhengzhou, 450015 China

**Keywords:** Crossflow, Water jet, High-speed photography, Jet penetration depth, Engineering, Physics, Fluid dynamics

## Abstract

The interaction between crossflow and liquid jets is common in engineering applications, such as in gas–steam catapult power systems and supersonic ramjets. Studying the atomization process of liquid jets in crossflow has significant engineering value. In this work, high-speed photography was used. The experimental results indicate that factors such as airflow velocity, temperature, jet velocity, temperature, and nozzle diameter can affect the depth of jet penetration. Considering the influence of various factors, an empirical formula for calculating jet penetration is obtained. The results can support the design of gas–steam catapult propulsion systems and be extended to other applications, such as supersonic ramjets.

## Introduction

The interaction between crossflow and liquid jets is prevalent in various engineering applications, such as gas–steam catapult power systems, supersonic ramjets, and film cooling protection. Studying the atomization mechanisms of liquid jets in crossflow holds significant academic importance and broad engineering application value.

The breakup process of liquid jets in crossflow is a complex two-phase turbulent gas–liquid process involving fluid dynamics, mass transfer, and heat transfer^1–4^, droplet breakup in the crossflow, and liquid evaporation^5–11^. Researchers have conducted studies of the motion trajectory of jets and the breakup of jet columns and droplets^12–14^.

Gao et al.^1^ experimentally studied the effect of cross-flow temperature (300 and 500 K) on the primary atomization of a liquid jet. The increase in the cross-flow temperature resulted in a slight increase in the column crushing height and near-field trajectory, and an empirical correlation for the near-field trajectory was obtained, including the effects of the cross-flow temperature and momentum flux ratio. Kasmaiee et al.^2^ studied the influence of the liquid jet injection angle on cross flow; and established a theoretical model of the liquid jet trajectory considering the influence of the jet angle by using high-speed photogrammetry and other parameters. Li et al.^6^ used the phase Doppler wind velocity method to conduct experimental research on the atomization characteristics of liquid jets in supersonic cross-flow. The jet cross sections of supersonic cross-flow jets are approximately ω shaped. The main influencing factor of droplet size is the aerodynamic force. Liu et al.^8^ proposed a simplified prediction model for the spatial distribution of droplets in transverse flows of subsonic gas; and measured the growth rate of surface disturbances in liquid columns via linear stability analysis method. Zhang and Zhou et al.^13,14^ reviewed the fragmentation mechanism, atomization characteristics and factors affecting the atomization of liquid jets in transverse airflow.

Most studies of liquid jets in crossflow have focused on the penetration depth, surface wave features, breakup location of the jet, and distributions of the gas and liquid phases and velocities. The penetration depth is a critical parameter for characterizing the breakup characteristics of liquid jets in crossflow. Some scholars^15–21^ have introduced parameters such as the momentum ratio into the empirical fitting formulas. The fitting relationships for penetration depth equations can be categorized into three forms: power function, logarithmic, and exponential. Despite extensive research, a unified fitting relationship has not been established.

Chang et al.^15^ studied the effects of the Weber number and momentum flux ratio (q) on subsonic cross-flow atomization; and analyzed the jet penetration depth, crushing position and droplet velocity distribution in detail. The larger q is, the stronger the penetration. Xie et al.^20^ established a comprehensive theoretical model of the initial rupture of a liquid jet in subsonic cross flow; and theoretically analyzed the deformation process of the jet, the mass stripping process and the influence of several critical forces, obtaining high-precision jet trajectory predictions. Zhang et al.^21^ studied the deflection deformation process of a circular jet in transverse shear airflow via numerical simulation, and obtained a trajectory curve formula by using the nozzle diameter and momentum ratio.

In the context of gas–steam catapult power systems, this paper focuses on the interaction between crossflow and water jets. In-depth research has been conducted on the development characteristics of the atomization of water jets given the effects of crossflow, and the penetration depth and the trajectory of the jets. The effects of different airflow, jet, and structural parameters on the macroscopic motion trends of water jets are investigated, via high-speed photography, to provide guidance for the design of gas–steam catapult power systems. The research findings can also be applied in other areas of study such as supersonic ramjets.

## Jet experimental scheme

### Experimental system

**Fig. 1:**
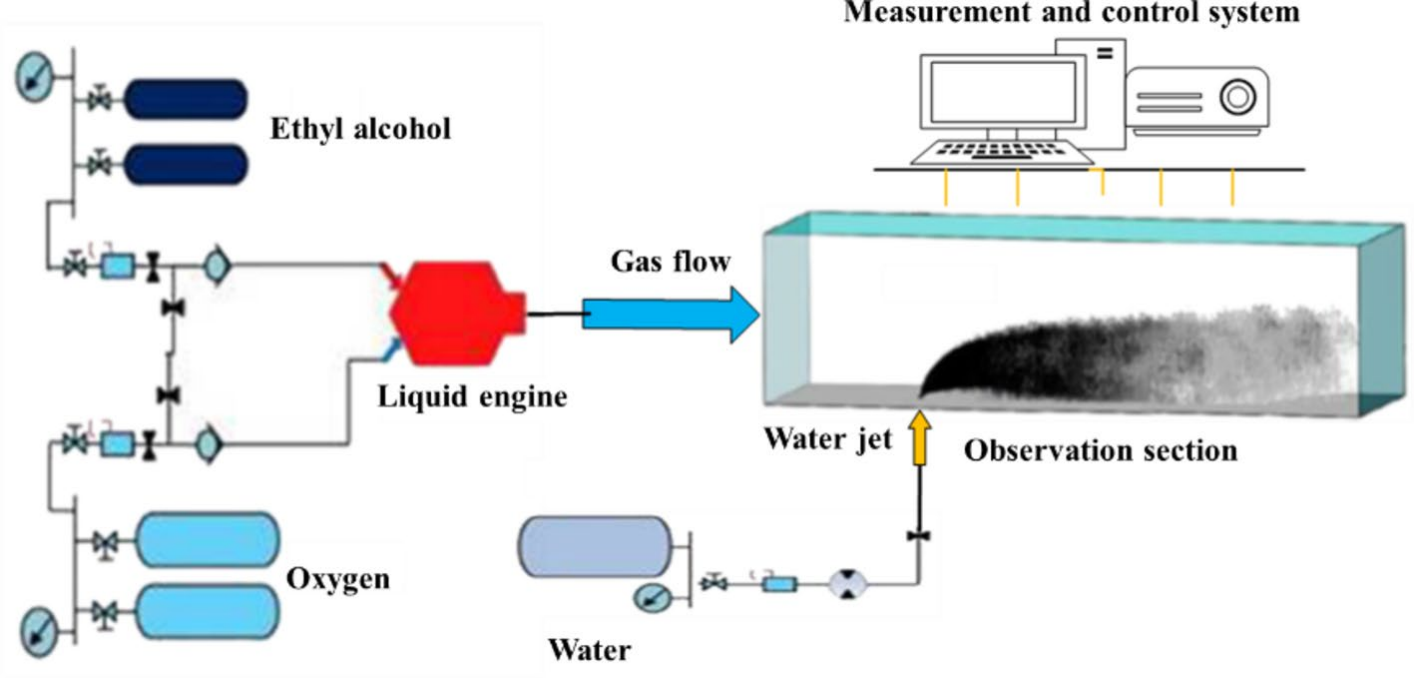
Diagram of the jet experimental system.

An experimental system for jets was designed on the basis of the atomization characteristics of a water jet in gas–steam catapult power system. This system mainly composed of a fuel and oxidizer, a supply system, a liquid engine, a jet flow system, an observation section and a measurement and control system, as shown in Fig. 1 .

Alcohol and oxygen are supplied into the liquid engine. The generated gas is accelerated through a Laval nozzle and enters the round-to-square section, with the cross-sectional shape of the flow path transformed to two dimensions, which is conducive to applying optical observation methods such as high-speed photography. After passing through the stabilization section, a uniform 2D airflow is obtained. The airflow enters the observation section, where liquid water is simultaneously sprayed from below. The liquid water breaks up and atomizes into small droplets under the effect of airflow and subsequently evaporates due to the high temperature.

The crossflow temperature and velocity are adjusted by changing the mixing ratio of alcohol and oxygen and replacing the nozzle sections with sections of different diameters, whereas the water jet flow and velocity are adjusted mainly by changing the injection pressure and the diameter of the nozzle.

**Fig. 2:**
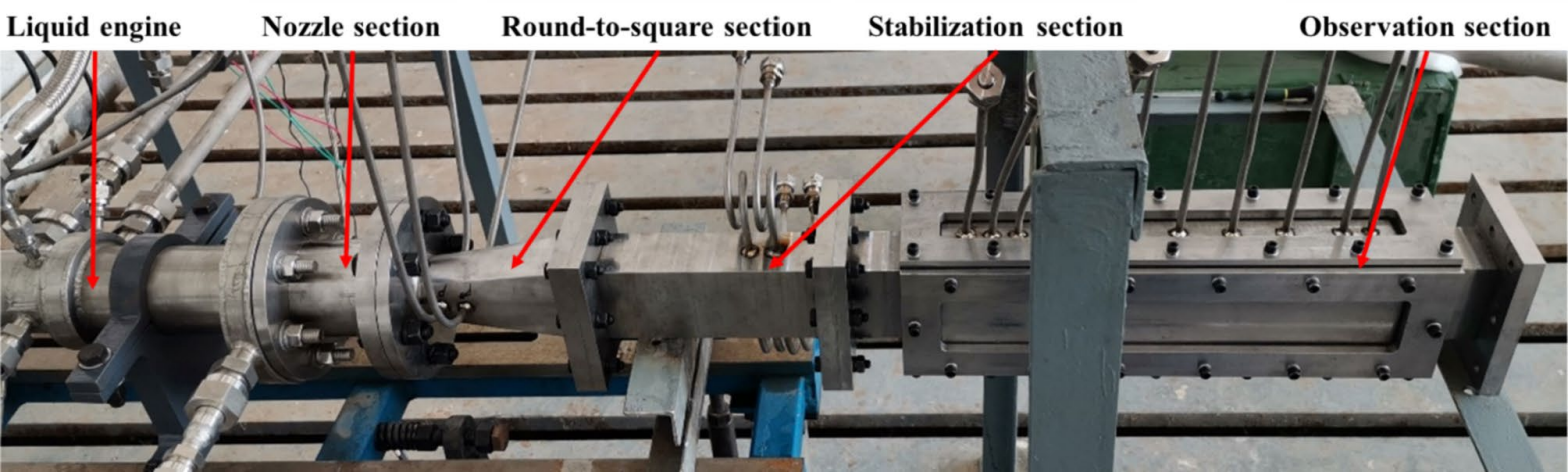
Jet atomization experimental system.

The jet atomization experimental system consists of a liquid engine, a round–to–square section, a stabilization section, and an observation section, as shown in Fig. 2. The nozzle section accelerates the gas to supersonic speeds. The round–to–square section transforms the circular nozzle into a square duct suitable for observation. After stabilization, high–speed camera observations are obtained in the observation section.

### Measurement system

High-speed photography has been widely used for capturing instantaneous images and transient processes. In this work, a Phantom Miro M340 high-speed camera is used to obtain the macroscopic structural features of the studied water jet. The MIRO M340 has 4 megapixels and can shoot at a maximum speed of 311,000 fps (frames/s), or 800 fps for 2560 × 1600 shots, with a minimum exposure time (shutter speed) of 1 µs and a minimum interval of 1.4 µs between exposures.

**Fig. 3:**
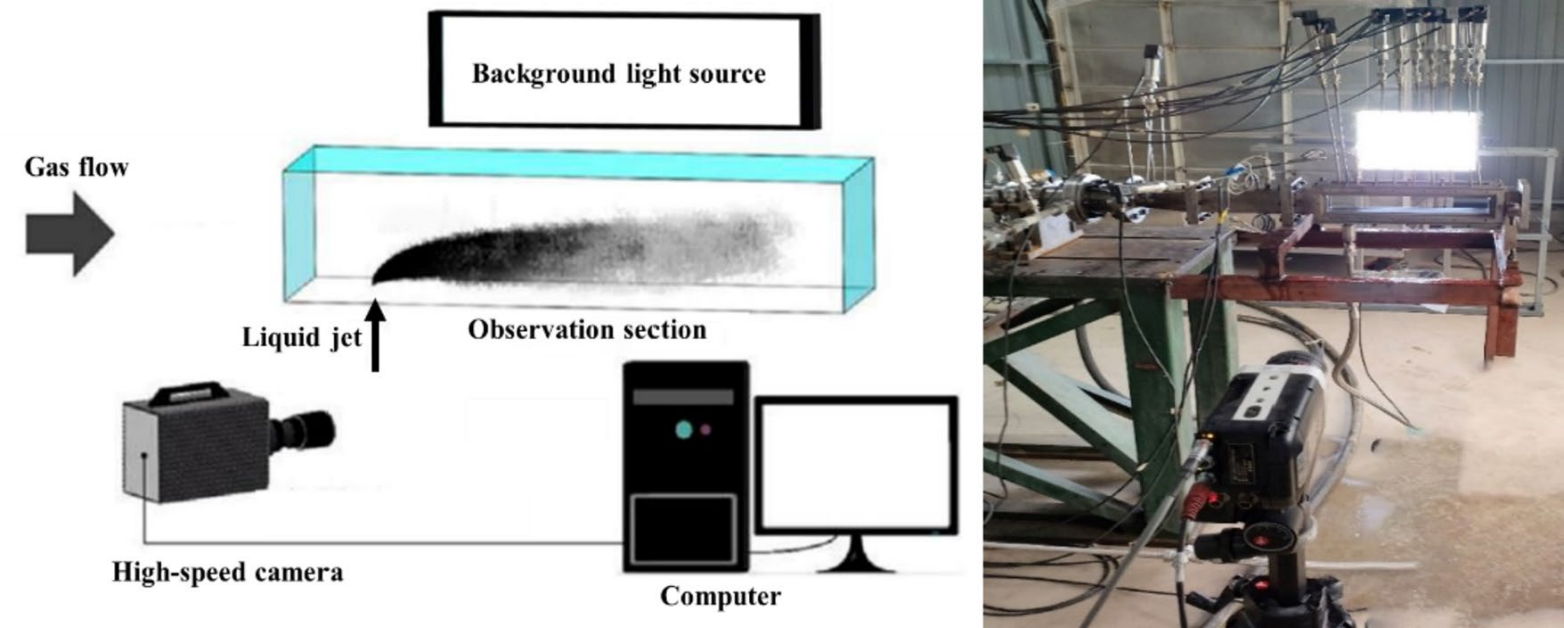
Relative position diagram of the high-speed camera and experimental system.

Figure 3 shows the relative positions of the high-speed camera and experimental system. During the experiment, the distance of the camera lens from the center plane of the observation section is approximately 1 m, and the precise focus of the high-speed camera on the water jet, which is controlled by a computer, is essential.

### Experimental conditions

On the basis of the characteristics of gas–steam catapult power systems, cold- and hot-state experimental studies are conducted to investigate the atomization of water jets. The specific experimental conditions are listed in Tables 1 and 2, with nine cold-state conditions and ten hot-state conditions. The subscript “gas” denotes the parameters at the inlet of the observation section, and the subscript “water” denotes the water jet parameters. Dt represents the throat diameter, D represents the nozzle diameter, We represents the Weber number, Ma represents the Mach number, q represents the momentum ratio, n represents the nozzle number, Ps0 represents the pressure in the water storage tank, and Ps1 represents the pressure before water jet ejection.

**Table 1:** Cold-state experimental conditions.

	T*_gas_/K	Dt/mm	U_gas_/m/s	We_gas_	Ma_gas_	T_water_/K	*P*_s0_/MPa	*P*_s1_/MPa	U_water_/m/s	D/mm	*n*	q
C–1	293	14.5	140	74	0.42	293	0.4	0.33	20	3	1	18.3
C–2	293	14.5	140	74	0.42	293	0.5	0.42	25	3	1	25.5
C–3	293	14.5	140	74	0.42	293	0.6	0.50	28.5	3	1	33.3
C–4	293	14.5	140	74	0.42	293	0.4	0.35	23.5	2	1	22.9
C–5	293	14.5	140	74	0.42	293	0.4	0.22	13	5	1	9.0
C–6	293	14.5	140	74	0.42	323	0.4	0.34	22.5	3	1	20.5
C–7	293	14.5	140	74	0.42	293	0.4	0.26	17.5	3	2	12.5
C–8	293	17.75	120	64	0.36	293	0.4	0.33	20	3	1	24.1
C–9	293	20.5	110	59	0.33	293	0.4	0.33	20	3	1	29.3

**Table 2:** Hot-state experimental conditions.

	T*_gas_/K	Dt/mm	U_gas_/m/s	We_gas_	Ma_gas_	*P*_s0_/MPa	*P*_s1_/MPa	U_water_/m/s	D/mm	q
H–1	1200	25.1	1000	200	1.85	1.6	1.35	50	3	5.5
H–2	1200	25.1	1000	200	1.85	1.1	0.9	40	3	3.5
H–3	1200	25.1	1000	200	1.85	2.1	1.8	60	3	7.5
H–4	1200	25.1	1000	200	1.85	1.1	1.0	45	2	4.3
H–5	1200	25.1	1000	200	1.85	2.1	1.9	65	2	8.9
H–6	1200	25.1	1000	200	1.85	1.6	1.45	55	2	6.5
H–7	1200	25.1	1000	200	1.85	1.6	0.65	33.5	5	2.8
H–8	1200	20.5	1050	235	1.99	1.6	1.35	50	3	4.2
H–9	1200	17.75	1100	255	2.15	1.6	1.35	50	3	3.8
H–10	2000	25.1	1250	180	1.91	1.6	1.35	50	3	4.4

### Analysis of experimental results

#### Development process of jet atomization

**Fig. 4:**
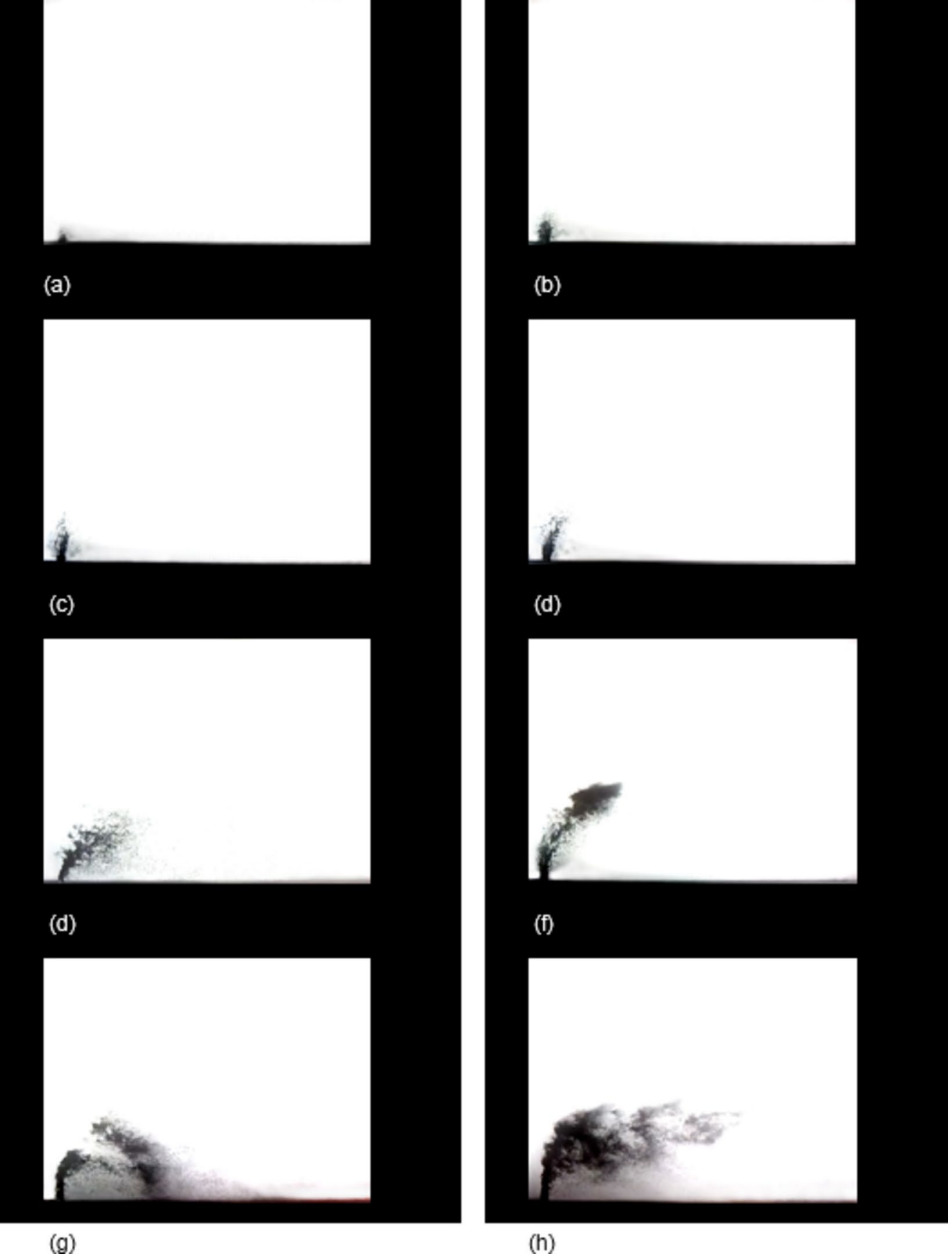
Development process of jet atomization.

The macroscopic morphology of the jet is analyzed via high-speed photography to capture the development and evolution process, as shown in Fig. 4. Initially, the water jet enters the crossflow with a high initial velocity, and there is minimal decay in the leading-edge velocity. The water jet maintains good vertical movement, as shown in Fig. 4 (a)-(d). As time progresses, the leading edge velocity decays, and the jet continuously experiences significant lateral shear forces, causing the cylindrical jet to bend. Large liquid cell structures peel off the surface of the jet column, and a low-pressure region forms on the leeward side, where noticeable liquid cell structures also detach, as depicted in Fig. 4 (e) -(f). As the jet further evolves, the detached liquid cell and filament structures break up into smaller liquid droplets, as shown in Fig. 4 (g)-(h).

### Factors influencing the jet penetration depth

#### Influence of the water jet velocity

**Fig. 5:**
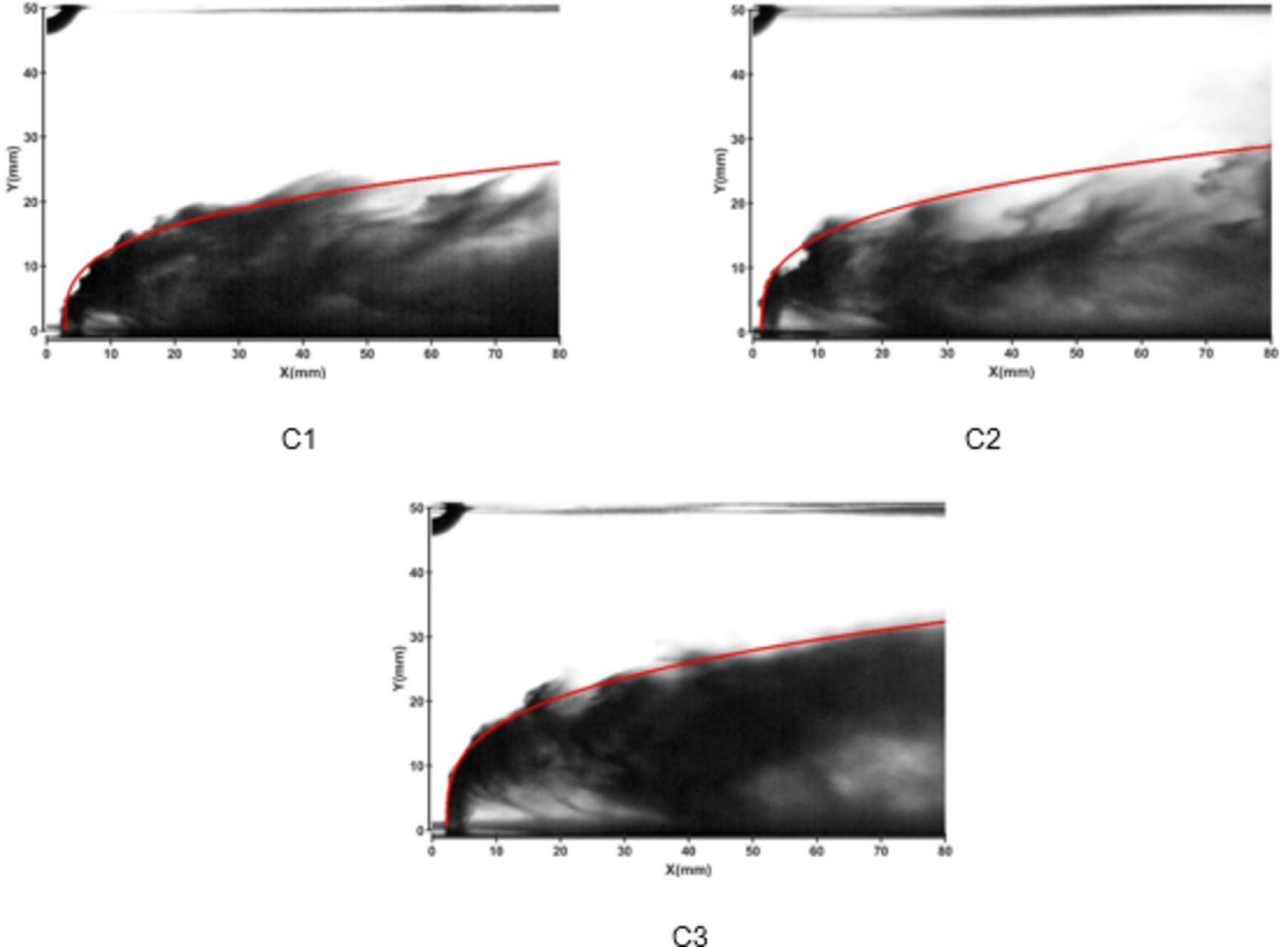
Jet development trajectories for experimental conditions C1, C2 and C3.

**Fig. 6:**
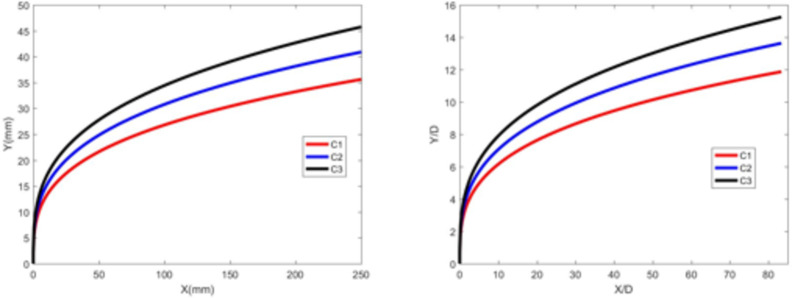
Theoretical curve of the jet penetration depth and the curve of the nondimensional nozzle diameter for experimental conditions C1, C2 and C3.

Figures 5 and 6 show the jet trajectories under different water jet velocities for experimental conditions C1, C2, and C3. By changing the pressure of the water storage tank, the water jet velocity can be altered. The jet trajectories for the three conditions are obtained, as shown in Fig. 5. The theoretical curve of the jet penetration depth and the curve of the nondimensional nozzle diameter are shown in Fig. 6. The figures show that the energy of the water jet increases with increasing water jet velocity, allowing it to penetrate deeper into the same crossflow.

**Fig. 7:**
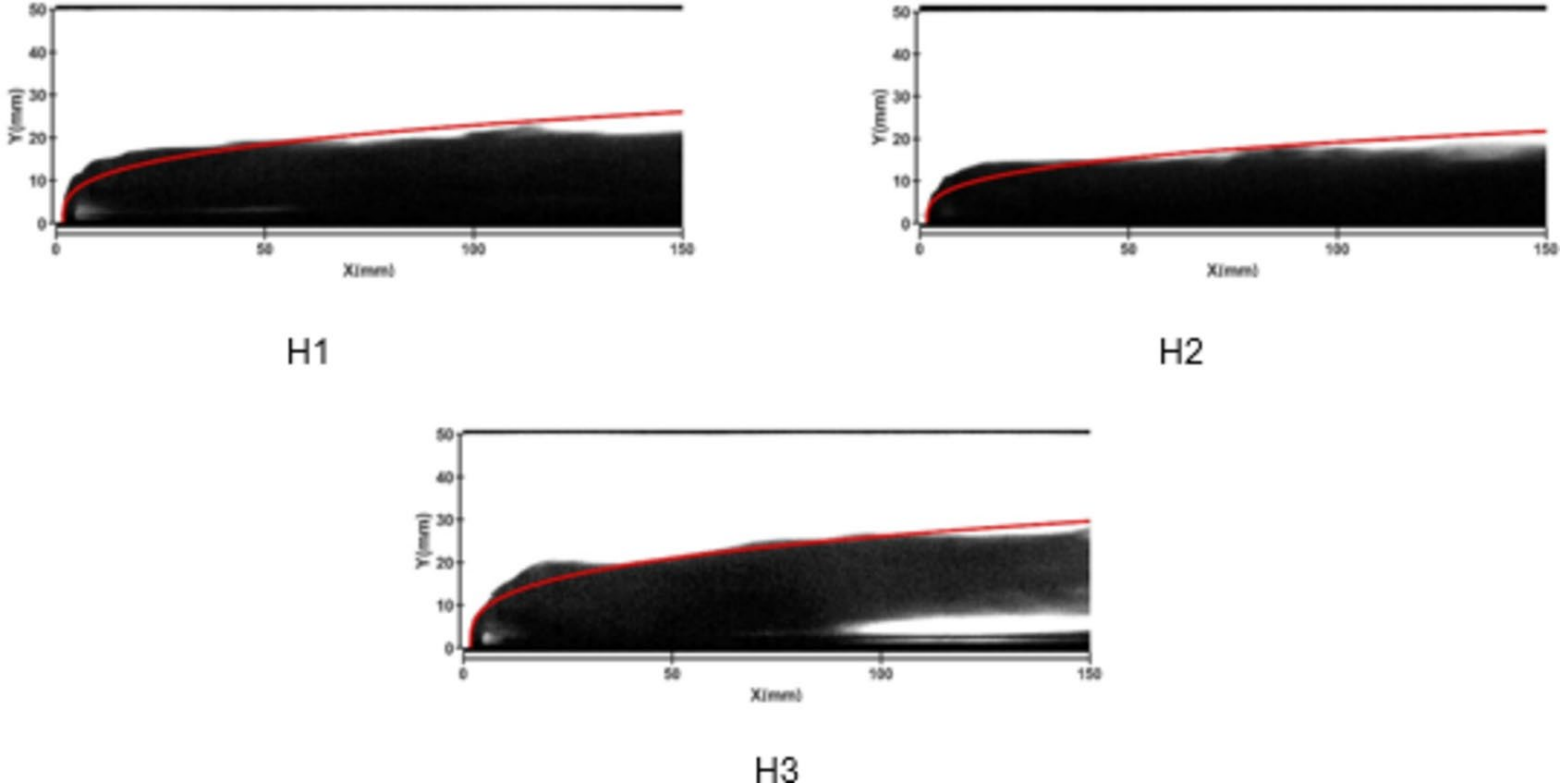
Jet development trajectories for experimental conditions H1, H2, and H3.

**Fig. 8:**
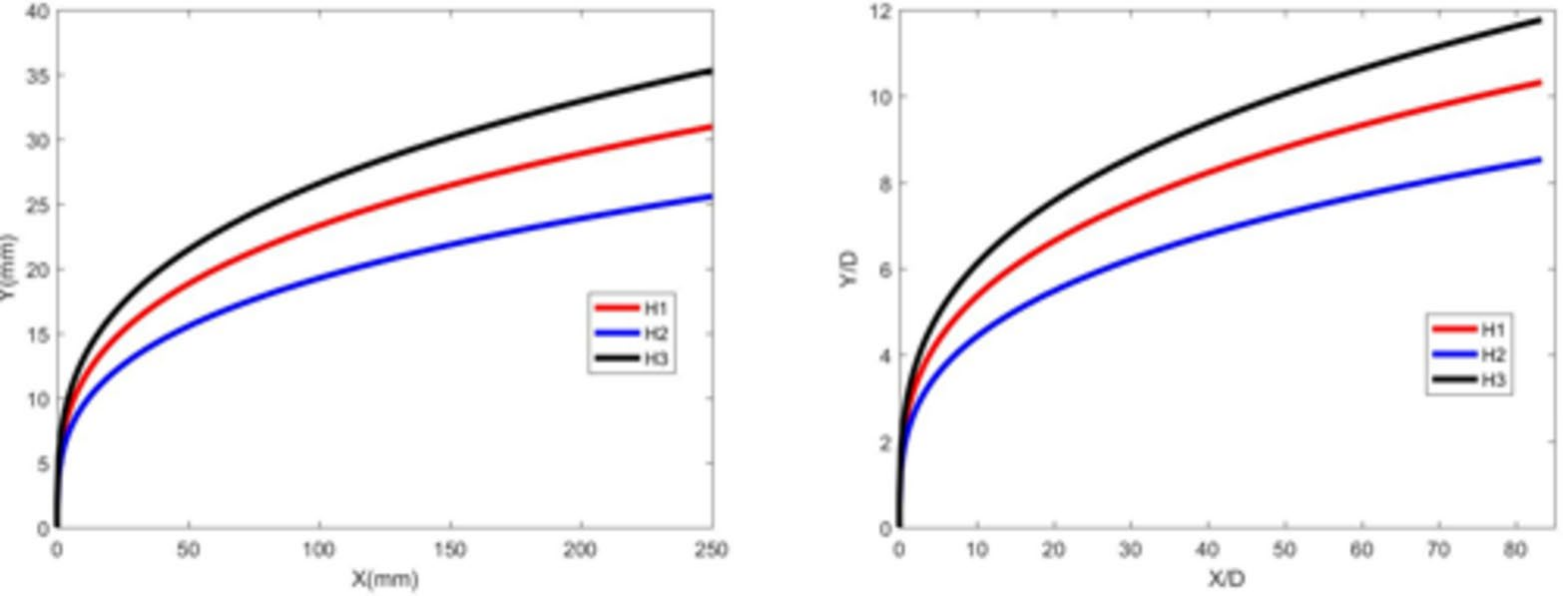
Theoretical curve of jet penetration depth and the curve of the nondimensional nozzle diameter for experimental conditions H1, H2, and H3.

**Fig. 9:**
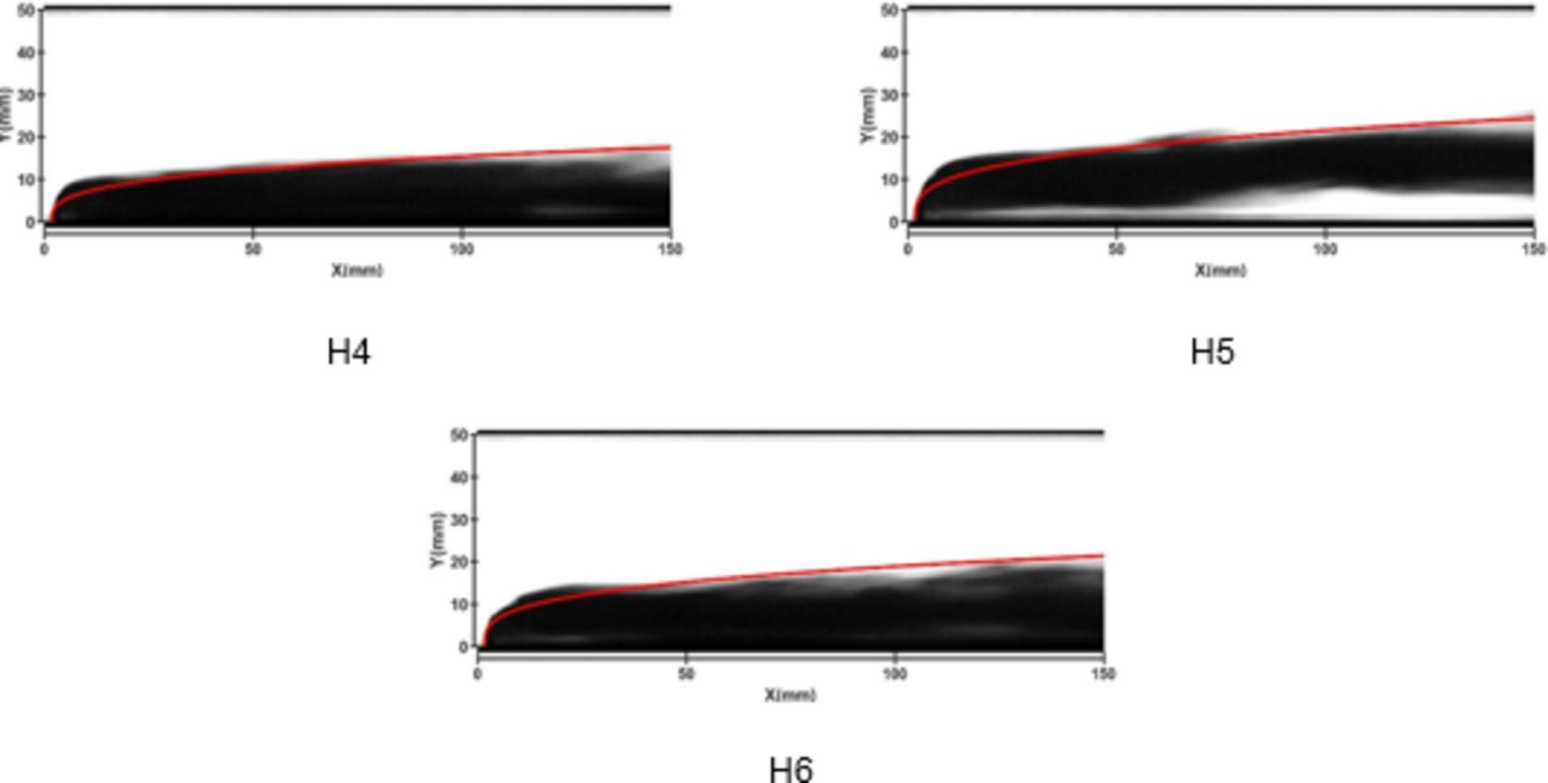
Jet development trajectories for experimental conditions H4, H5 and H6.

**Fig. 10:**
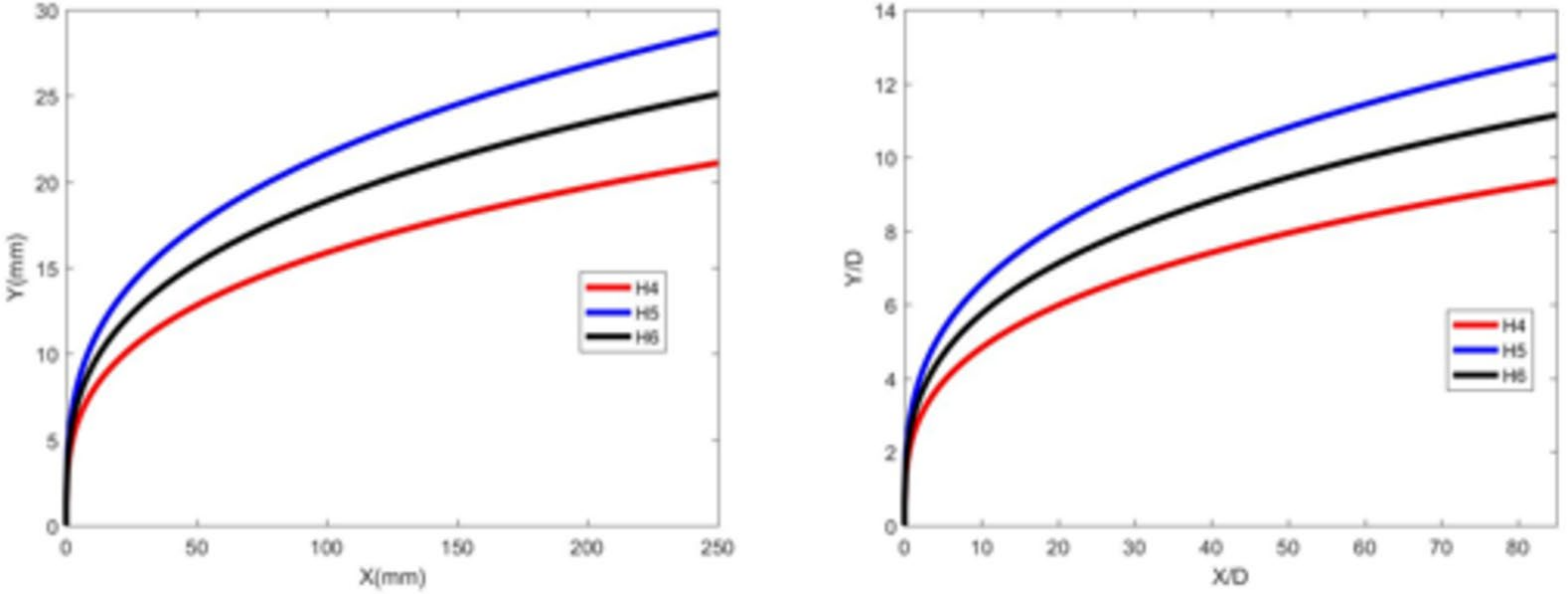
Theoretical curve of jet penetration depth and the curve of the nondimensional nozzle diameter for experimental conditions H4, H5 and H6.

Figures 7 and 8 depict the jet trajectories for different water jet velocities under experimental conditions with a 3-mm nozzle diameter. Figures 9 and 10 show the jet trajectories for a 2-mm nozzle diameter. By changing the pressure of the water storage tank, the water jet velocity can be altered. The jet trajectories are obtained, as shown in Figs. 7 and 9. The theoretical curve of the jet penetration depth and the curve of the nondimensional nozzle diameter are shown in Figs. 8 and 10. An increase in the water jet velocity leads to an increase in the energy of the water jet, allowing it to penetrate deeper into the same crossflow. The trend for the 3 -mm nozzle diameter is consistent with that for the 2 -mm nozzle diameter and that under cold-state experimental conditions.

### Influence of nozzle diameter

**Fig. 11:**
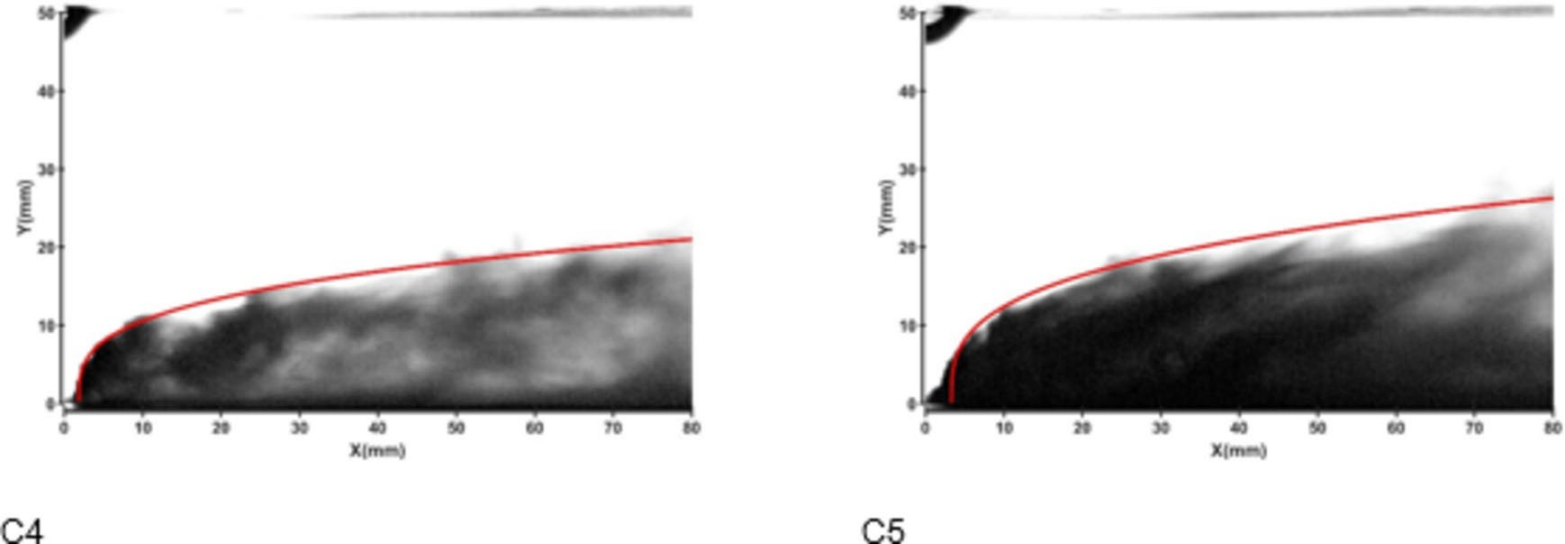
Jet development trajectories for experimental conditions C4 and C5.

**Fig. 12:**
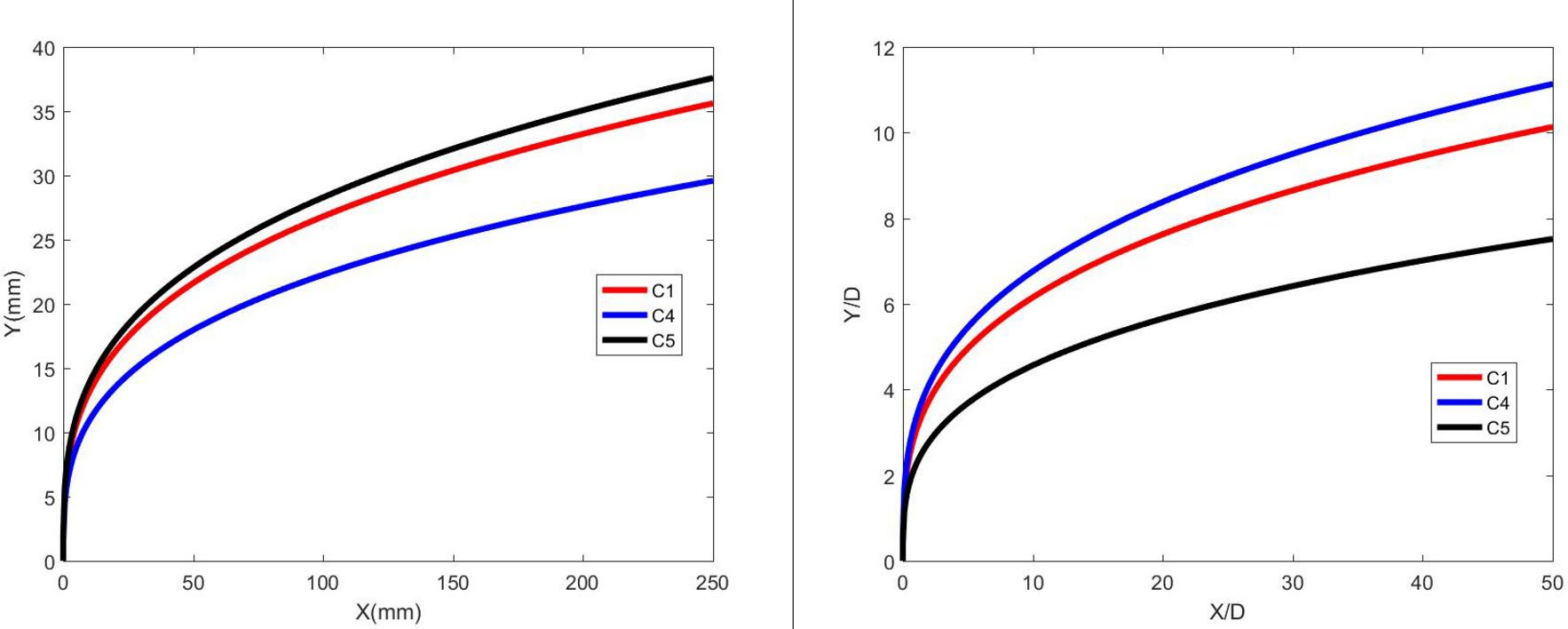
Theoretical curve of the jet penetration depth and the curve of the nondimensional nozzle diameter for experimental conditions C1, C4 and C5.

Figures 11 and 12 illustrate the development of jet trajectories under different nozzle diameters for experimental conditions C4 and C5. The jet trajectories were obtained, as shown in Fig. 11. The theoretical curve of the jet penetration depth and the curve of the nondimensional nozzle diameter are shown in Fig. 12. The figures show that the jet penetration depth increases with increasing nozzle diameter, whereas the dimensionless jet penetration depth has the opposite effect. Given that the pressure in the water jet storage tank is contant, the energy of the water jet remains approximately the same. A reduction in nozzle diameter results in a higher initial velocity and a smaller initial deflection angle for the jet. However, a smaller nozzle diameter equates to lower flow rate, causing the jet to decay faster, thereby leading to a smaller penetration depth. Conversely, a larger nozzle diameter leads to a greater penetration depth.

**Fig. 13:**
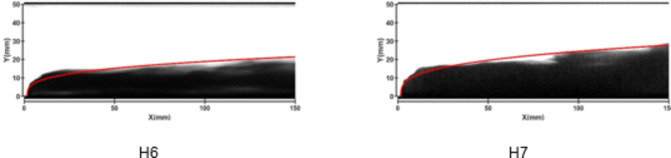
Jet development trajectories for experimental conditions H6 and H7.

**Fig. 14:**
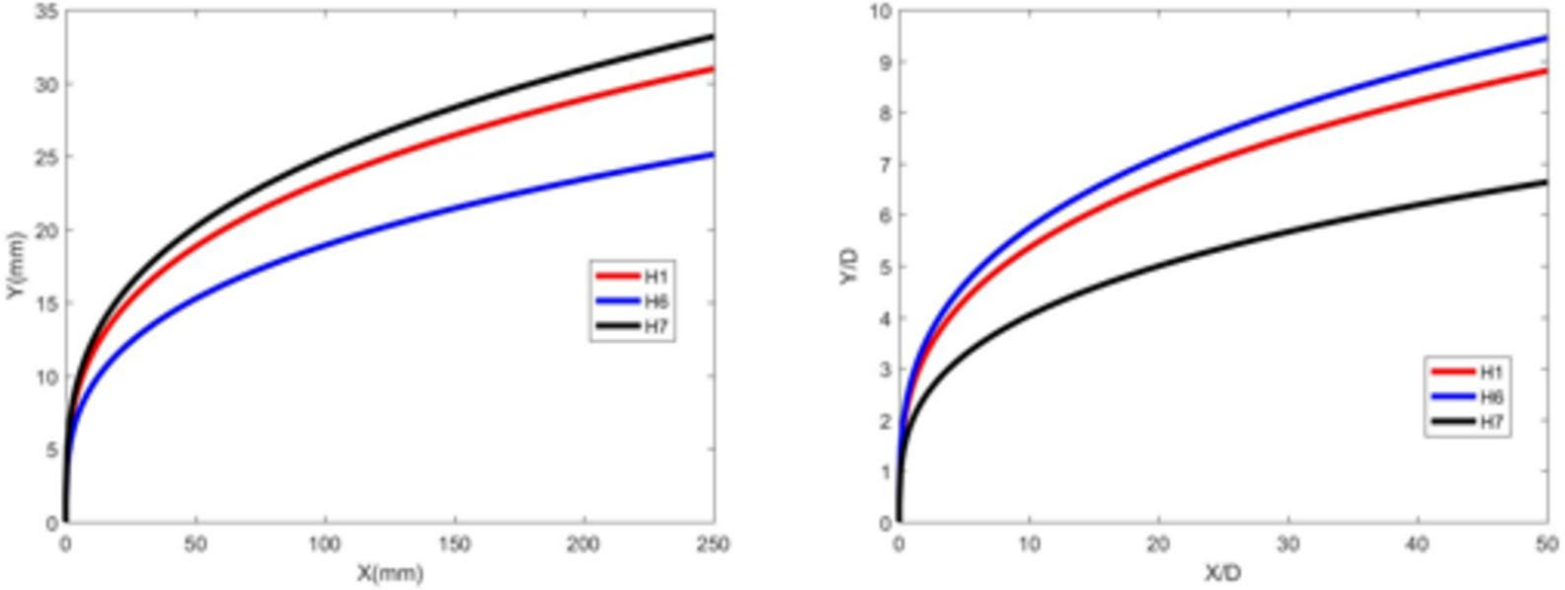
Theoretical curve of the jet penetration depth and the curve of the nondimensional nozzle diameter for experimental conditions H1, H6 and H7.

Figures 13 and 14 depict the development of jet trajectories under different nozzle diameters for experimental conditions H6 and H7. The jet trajectories are obtained, as shown in Fig. 13. The theoretical curve of the jet penetration depth and the curve of the nondimensional nozzle diameter are shown in Fig. 14. The figures show that the jet penetration depth increases with increasing nozzle diameter, whereas the dimensionless jet penetration depth has the opposite effect. This trend is consistent with the result observed under cold-state test conditions.

### Influence of water temperature

**Fig. 15:**
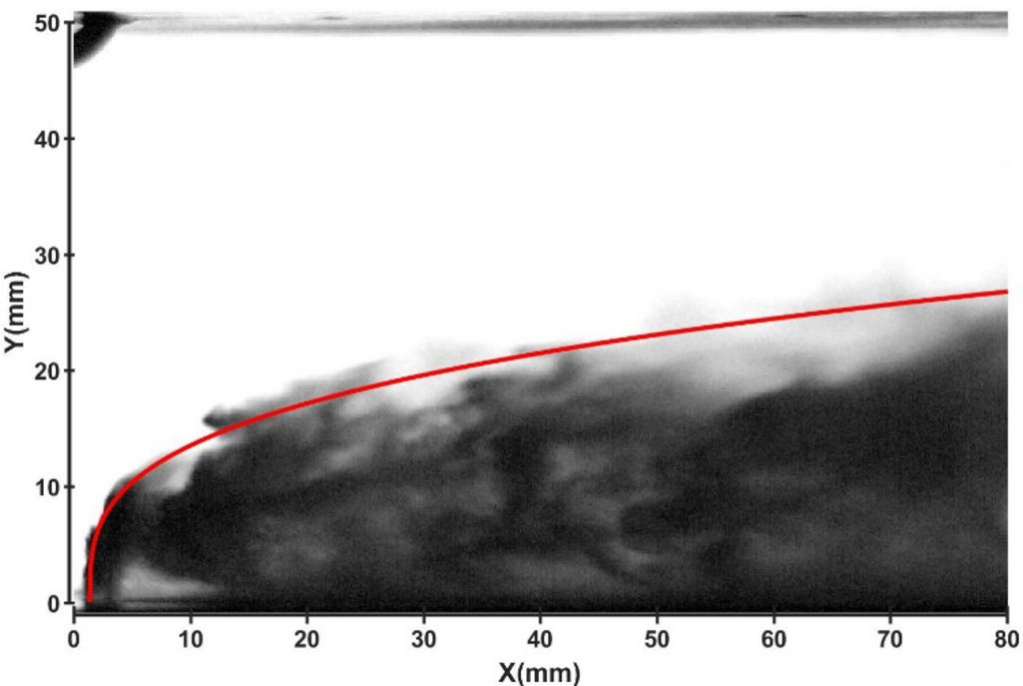
Jet development trajectory for experimental condition C6.

**Fig. 16:**
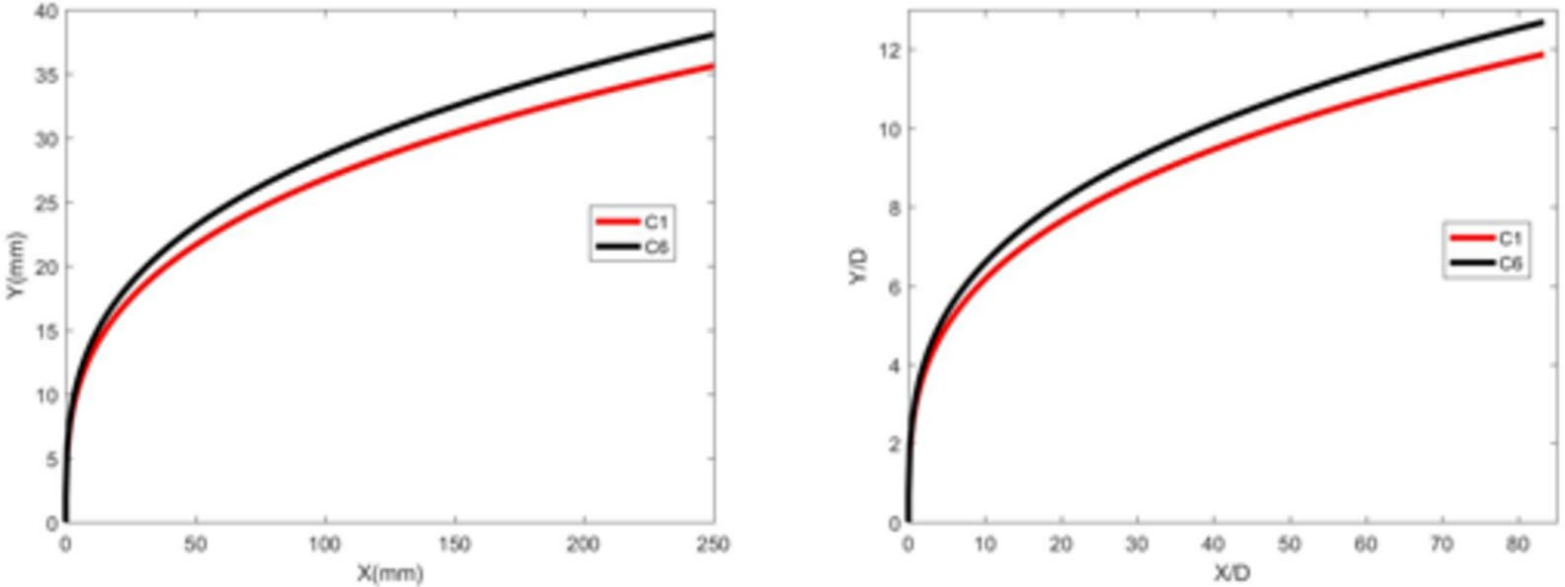
Theoretical curve of the jet penetration depth and the curve of the nondimensional nozzle diameter for experimental conditions C1 and C6.

Figures 15 and 16 show the development of jet trajectories under different water jet temperatures for experimental condition C6. The jet trajectories are obtained, as shown in Fig. 15. The theoretical curve of the jet penetration depth and the curve of the nondimensional nozzle diameter are shown in Fig. 16. From the figures, the jet trajectories for the experimental conditions are relatively similar. Compared with that under low-temperature conditions, the penetration depth under high-temperature water jet experimental conditions slightly increases.

### Influence of the number of nozzles

**Fig. 17:**
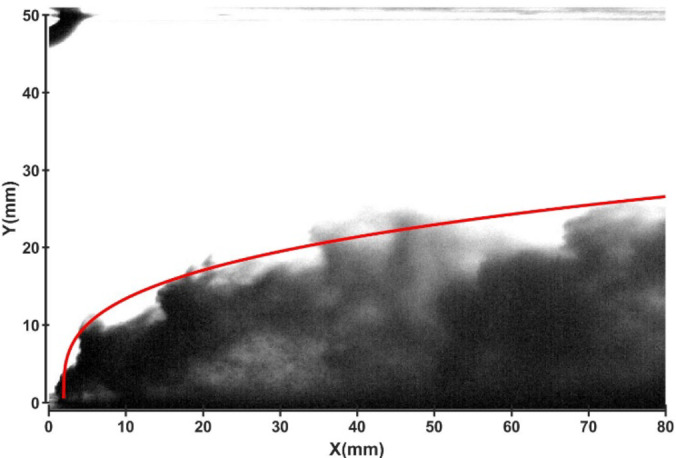
Jet development trajectory for experimental condition C7.

**Fig. 18:**
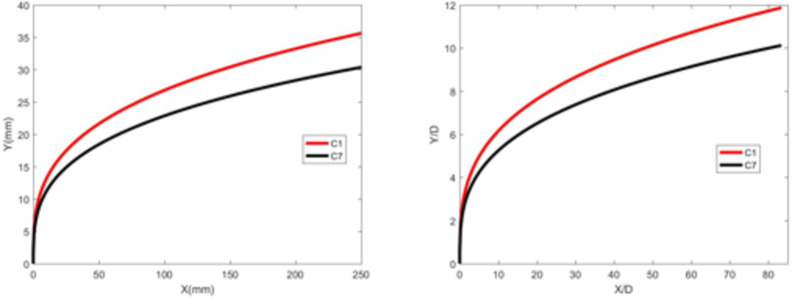
Theoretical curve of the jet penetration depth and the curve of the nondimensional nozzle diameter for experimental conditions C1 and C7.

Figures 17 and 18 illustrate the development of jet trajectories for cases with different numbers of nozzles for experimental condition C7. The jet trajectories are obtained, as shown in Fig. 17. The theoretical curve of the jet penetration depth and the curve of the nondimensional nozzle diameter are shown in Fig. 18. From the figures, an increase in the number of nozzles leads to a decrease in the water jet injection pressure, and a decrease in the water jet velocity, thus resulting in a smaller penetration depth. For the multiple-nozzle experimental conditions, the initial deflection angle of the jet from the rear nozzles is smaller than that under single-nozzle conditions because of the blocking effect of the jets from the front nozzles on the crossflow, but the overall penetration depth is not significantly affected.

### Influence of the crossflow velocity

**Fig. 19:**
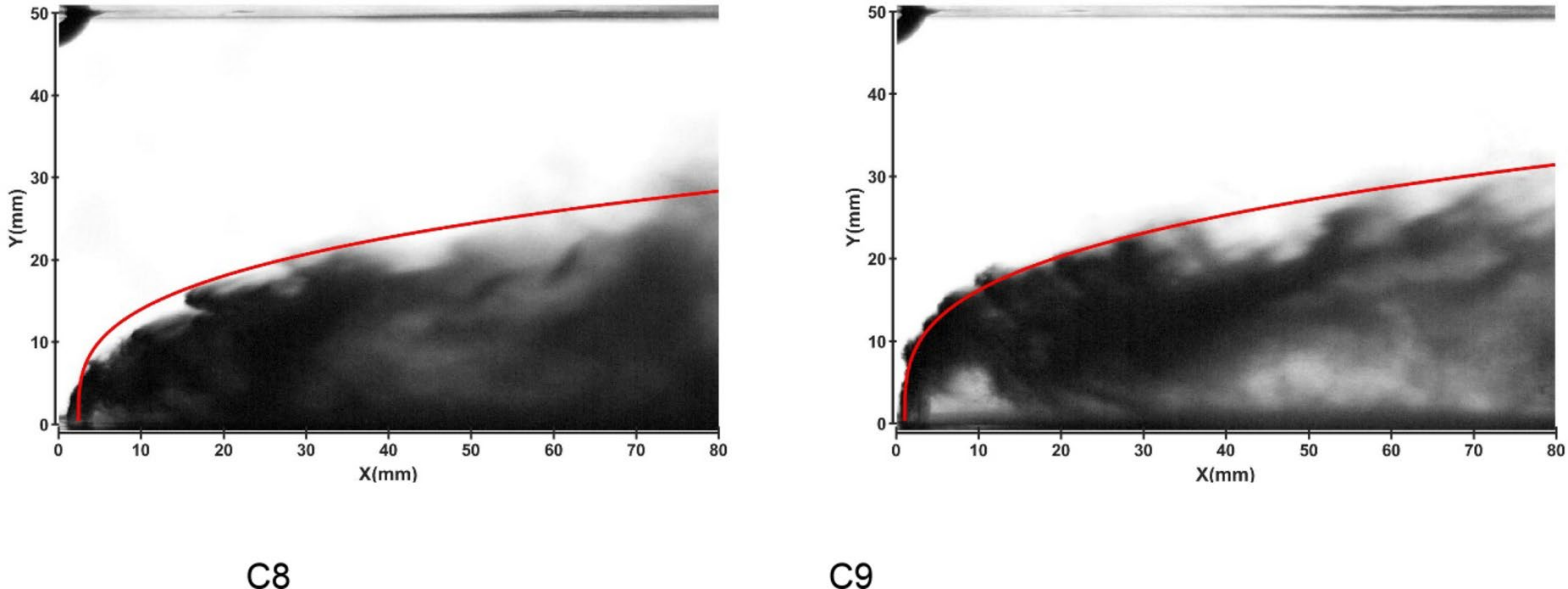
Jet development trajectories for experimental conditions C8 and C9.

**Fig. 20:**
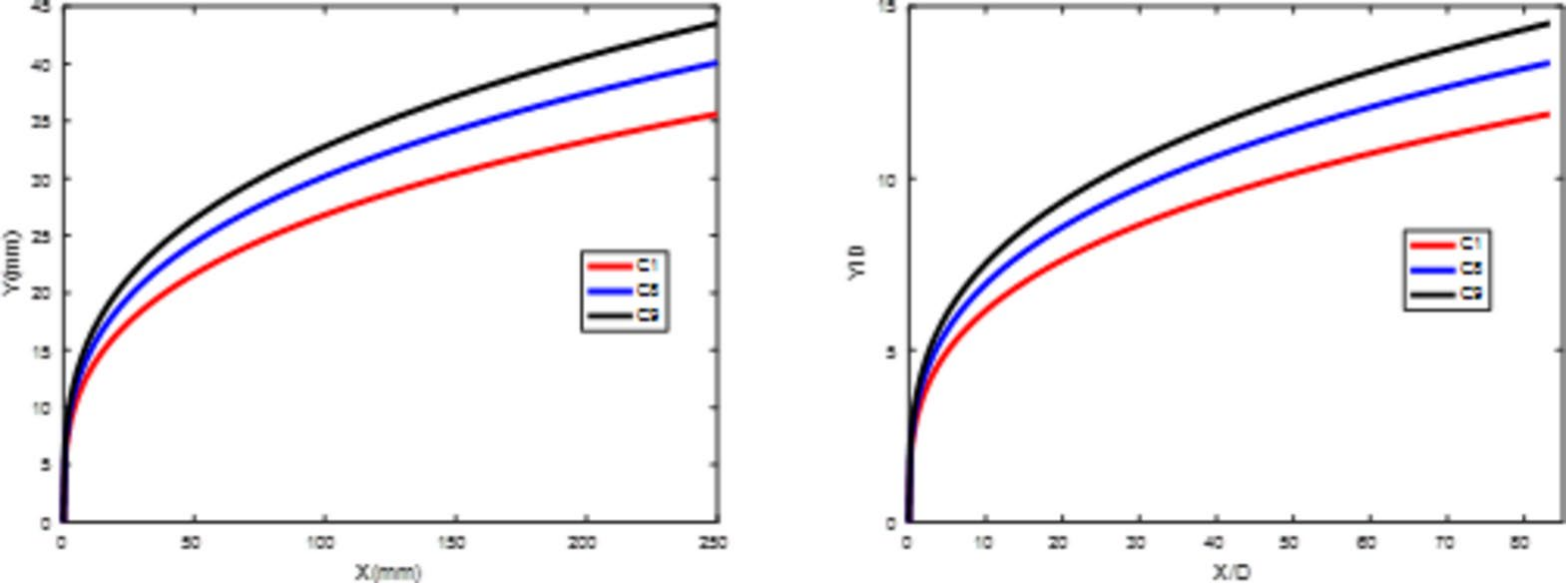
Theoretical curve of the jet penetration depth and the curve of the nondimensional nozzle diameter for experimental conditions C1, C8 and C9

Figures 19 and 20 depict the development of jet trajectories under different crossflow velocities for experimental conditions C8 and C9. The jet trajectories are obtained, as shown in Fig. 19. The theoretical curve of the jet penetration depth and the curve of the nondimensional nozzle diameter are shown in Fig. 20. From the figures, a decrease in the crossflow velocity reduces the obstruction of the water jet, thus preserving jet momentum, leading to an increase in the depth of jet penetration.

Figure 20 Theoretical curve of the jet penetration depth and the curve of the nondimensional nozzle diameter for experimental conditions C1, C8 and C9.

**Fig. 21:**
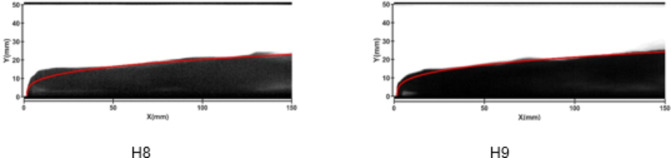
Jet development trajectories for experimental conditions H8 and H9.

**Fig. 22:**
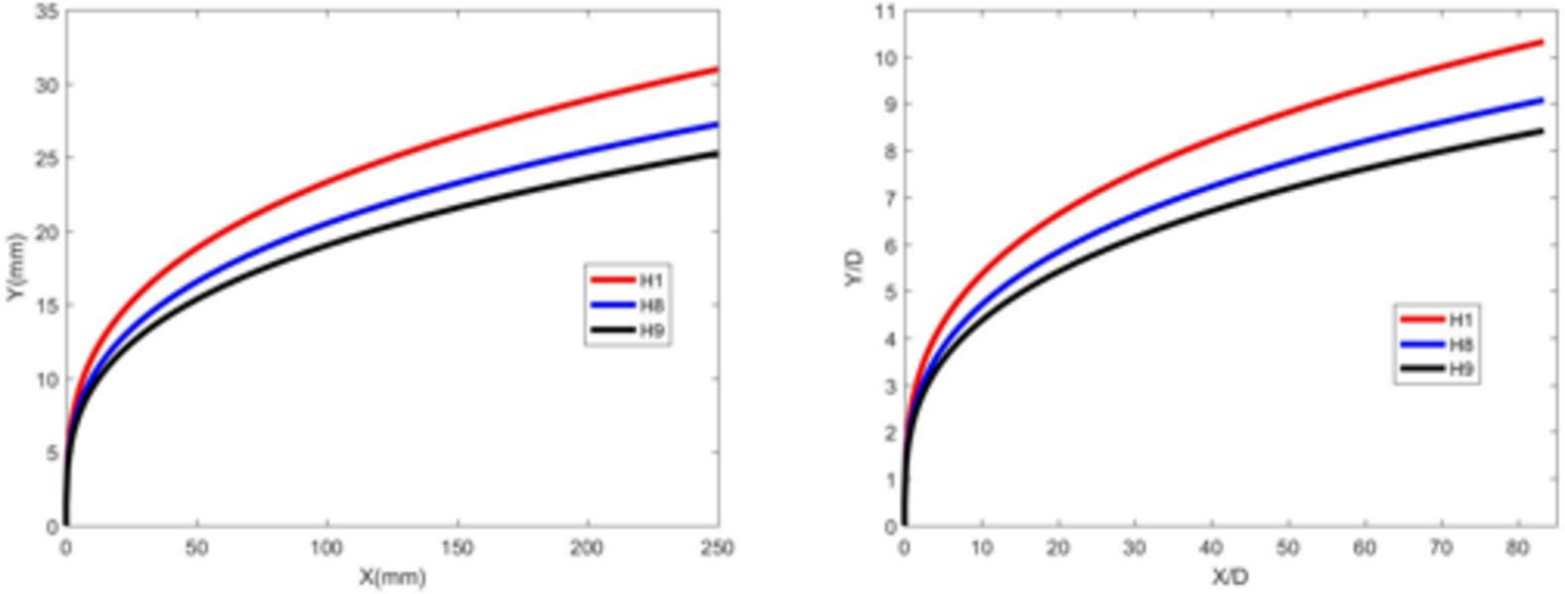
Theoretical curve of the jet penetration depth and the curve of the nondimensional nozzle diameter for experimental conditions H1, H8 and H9.

Figures 20 and 22 illustrate the development of jet trajectories under different crossflow velocities for experimental conditions H8 and H9. The jet trajectories are obtained, as shown in Fig. 21. The theoretical curve of the jet penetration depth and the curve of the nondimensional nozzle diameter are shown in Fig. 22. From the figures, a decrease in the crossflow velocity reduces the obstruction of the water jet, thus preserving momentum, leading to an increase in the depth of jet penetration. This trend is consistent with that observed during the cold-state experiment.

### Influence of the crossflow temperature

**Fig. 23:**
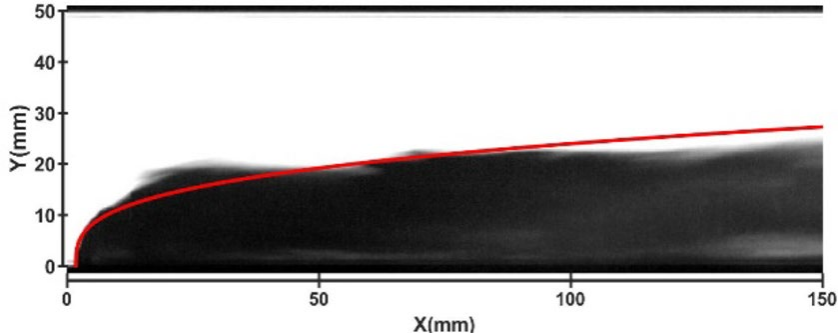
Jet development trajectory for experimental condition H10.

**Fig. 24:**
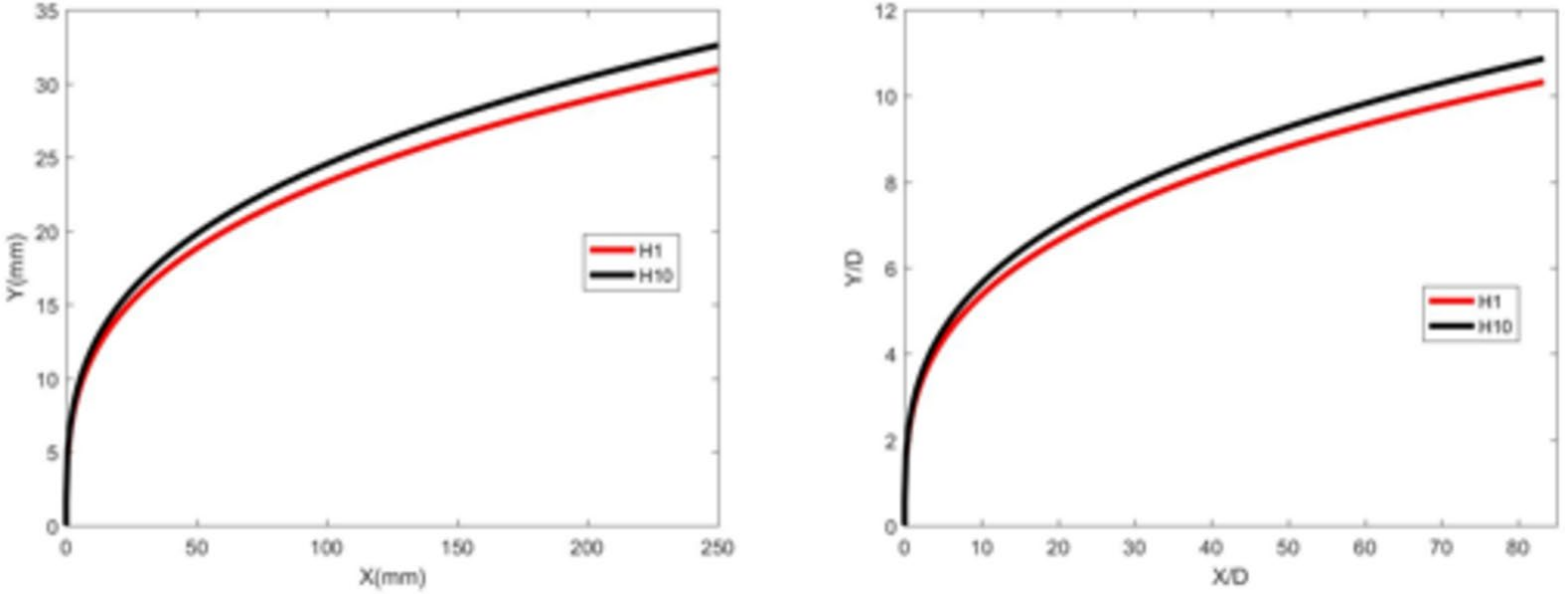
Theoretical curve of the jet penetration depth and the curve of the nondimensional nozzle diameter for experimental conditions H1 and H10.

Figures 22 and 23 show the jet trajectories under different crossflow temperatures for experimental conditions H1 and H10. The jet trajectories are obtained, as shown in Fig. 22. The theoretical curve of the jet penetration depth and the curve of the nondimensional nozzle diameter are shown in Fig. 24. As the temperature of the airflow increases, the corresponding airflow velocity also increases. The combined effect of these two parameters on the jet trajectory is complex, leading to a decrease in the depth of jet penetration. The specific influence of the crossflow temperature on the jet penetration depth requires further study.

**Fig. 25:**
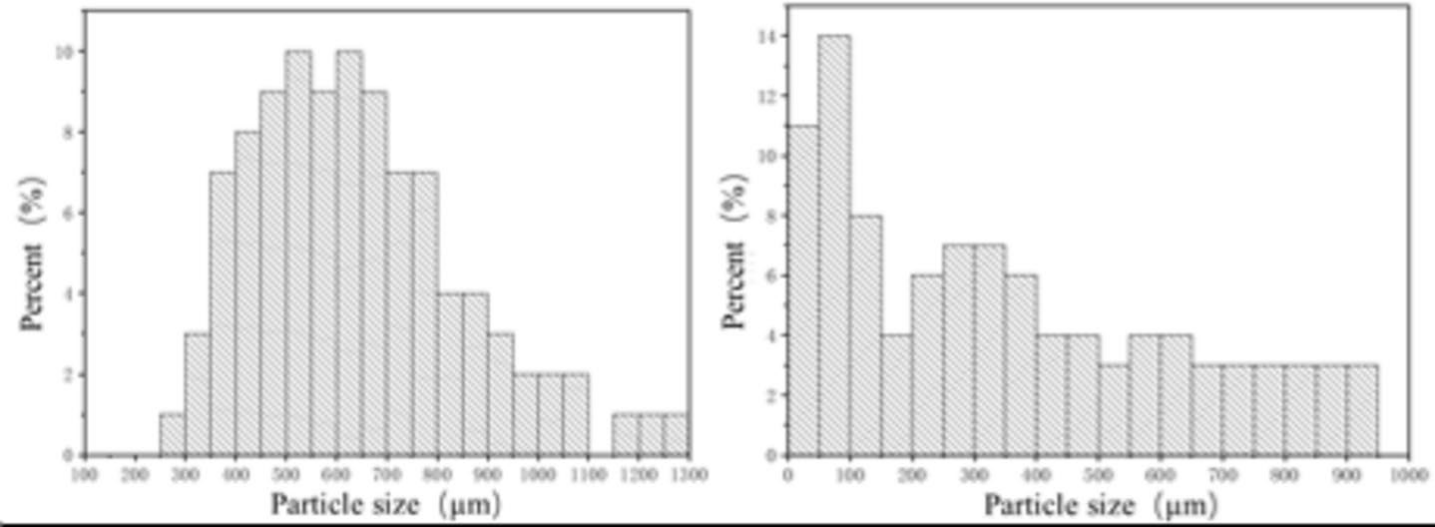
Particle size distributions of the droplets under experimental conditions C1 and H1.

A laser particle size analyzer was used to measure the droplet particle size of the spray in the flow field. The droplet particle size distributions under conditions C1 and H1 are shown in Fig. 25. The figure shows that the droplet particle size ranges from 250 to 1300 μm under cold conditions, whereas the droplet particle size ranges from 0 to 950 μm under hot conditions, thus the particle size under hot conditions is smaller than that under cold conditions.

**Fig. 26:**
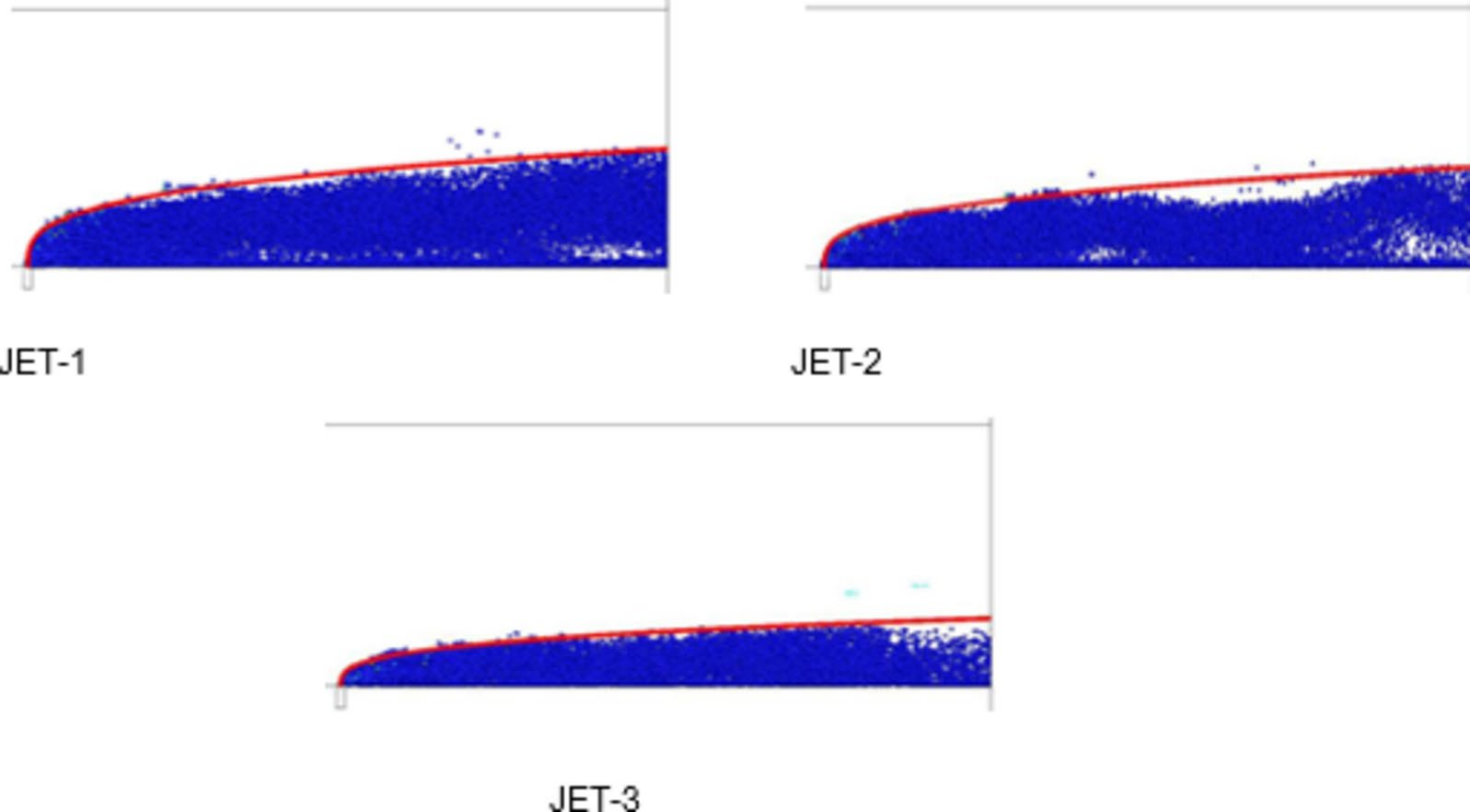
Jet development trajectory for simulation conditions JET-1, JET-2 and JET-3.

The simulation calculation under different air temperature conditions is carried out, and the jet movement trajectory is obtained as shown in Fig. 26. The temperatures of JET-1, JET-2 and JET-3 are 3000, 2500 and 2000 K, respectively, and the other parameters are the same. As shown in the figure, the jet penetration depth gradually decreases with decreasing air flow temperature. With decreasing air flow temperature, increasing air flow density and decreasing liquid-gas momentum ratio, the obstruction effect on the liquid jet increases, and the penetration depth of the jet decreases.

### Theoretical model of the jet penetration depth

The jet trajectory is a critical parameter for the development and evolution of jets, and is closely related to the jet and crossflow velocities. In crossflow, the water jet is subjected to aerodynamic forces, causing the continuous liquid column to bend.

**Fig. 27:**
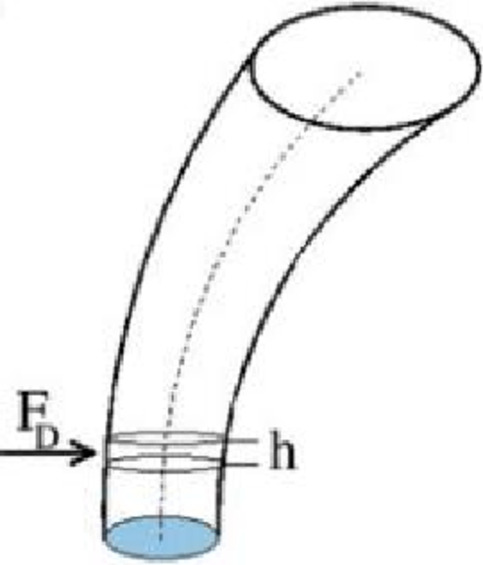
Schematic of the microcylindrical elements of the jet.

A cylindrical jet can be simplified as a simple model consisting of microcylindrical elements of diameter *d* and thickness *h*, as shown in Fig. 27. The following assumption can be made to derive the general form of the theoretical curve for the jet penetration depth:

(1) No viscous forces act between the micro cylindrical elements of the jet.

(2) No shear breakup or droplet detachment occurs during the development of the jet, and no mass is lost from the liquid column.

(3) The shape of the jet does not change during its development, and the cross-section of the liquid column remains circular.

The micro cylindrical elements of the jet are subjected to the aerodynamic force *F*_*D*_, which is expressed as:

1 $$F_{D}=\frac{1}{2}\rho_{8}u_{8}^{2}A_{F}C_{d}$$

where *A*_*F*_ is the windward area of the micro cylindrical element; hence *A*_*F*_ = *d* × *h*, and *C*_*d*_ is the drag coefficient.

To simplify the model, the viscosity and gravity of the micro cylindrical element are neglected, and the jet is treated as an ideal cylinder that does not deform with the motion of the jet. The acceleration of the microcylindrical elements along the direction of the crossflow is given by:

2 $$\frac{1}{2}\rho_{8}u_{8}^{2}A_{F}C_{d}=\rho_{l}A_{c}h\frac{d^{2}x}{dt^{2}}$$

where *A*_*C*_ is the cross-sectional area and *A*_*C*_ = *πd²/4*. Substituting *A*_*F*_ and *A*_*C*_ into the equation and integrating it twice yields:

3 $$\frac{x}{d}=\frac{1}{\pi}C_{d}\frac{\rho_{g}}{\rho_{l}}u_{s}^{2}\frac{t^{2}}{d^{2}}$$

Assuming that the microcylindrical elements of the jet have no momentum in the direction of the crossflow after exiting the nozzle, time *t* can be expressed as:

4 $$t=\frac{y}{u_{l}}$$

Substituting the expression for *t* into the derived equation yields a relationship without time terms:

5 $$\frac{x}{d}=\frac{1}{\pi}C_{d}\frac{\rho_{g}}{\rho_{l}}\left(\frac{u_{g}}{u_{l}}\right)^{2}\left(\frac{y}{d}\right)^{2}$$

Thus, the theoretical formula for the jet penetration depth curve under these assumptions is as follows^12^:

6 $$\frac{y}{d}=Cq^{\alpha}\left(\frac{x}{d}\right)^{\beta}$$

where *C*, *α*, and *β* are undetermined constants.

Although the assumptions made in the derivation process may not be entirely appropriate, the relationship above indicates that the jet penetration depth is significantly influenced by the momentum ratio of the jet and the airflow. This form of the equation is commonly used form among scholars domestically and internationally^12^. The empirical formulas for the jet penetration depth in this paper have the same general form as the above formula, but also consider the influence of factors such as the momentum ratio, and nozzle diameter. The development process of the jet penetration depth along the air flow direction under each set of experimental conditions is explored, and the value of each undetermined coefficient is calculated via jet penetration depth equation under different experimental conditions according to the number of undetermined parameters. The jet penetration depth is estimated by averaging the upper boundary values of the jet trajectory. Finally, the following fitting relationship for the jet penetration depth is obtained:

7 $$\frac{y}{d}=0.9{q^{0.42}}{\left( {\frac{x}{d}} \right)^{0.31}}{\left( {\frac{{{T_g}}}{{{T_0}}}} \right)^{0.37}}{\left( {\frac{{{T_l}}}{{{T_0}}}} \right)^{0.2}}$$

where *d* is the nozzle diameter; *q* is the momentum ratio; *y* is the magnitude of flow in the water jet direction; *x* is the magnitude of flow in the crossflow direction; *T*_*g*_ is the crossflow temperature; *T*_*l*_ is the water jet temperature; and *T*_*0*_ is the reference temperature of 293 K.

## Conclusion

In this work, high-speed photography was used to study the development and evolution process of a water jet under various operating conditions. The following conclusions were obtained:

1. In this study, a jet experimental system was established. High-speed photography was used to observe the macroscopic characteristics of the interaction field between the water jet and crossflow.
2. Factors such as the inflow velocity, temperature, jet velocity, temperature, and nozzle diameter all influence the development and evolution of the water jet.
3. Considering factors such as the momentum ratio, and nozzle diameter, the jet penetration depth fitting formula was obtained as $\frac{y}{d}=0.9{q^{0.42}}{\left( {\frac{x}{d}} \right)^{0.31}}{\left( {\frac{{{T_g}}}{{{T_0}}}} \right)^{0.37}}{\left( {\frac{{{T_l}}}{{{T_0}}}} \right)^{0.2}}$. This study provides guidance for the design of gas–steam catapult power systems, and its findings can be applied in other areas, such as the design of scramjet engines.

## Data Availability

The datasets used and/or analysed during the current study available from the corresponding author on reasonable request.
